# Effects of phytoestrogens combined with cold stress on sperm parameters and testicular proteomics in rats

**DOI:** 10.1515/biol-2022-0531

**Published:** 2023-01-24

**Authors:** Zhang Panpan, Nurbiah Alifu, Meiheriguli Sataer, Adilijiang Yiming, Siyiti Amuti, Ma Wenjing, Wang Binghua

**Affiliations:** School of Pharmacy, Xinjiang Medical University, Urumqi, China; Department of Human Anatomy, School of Basic Medical Sciences, Xinjiang Medical University, Shangde North Road 345, Urumqi 830017, China; Infirmary of Xinjiang Medical University, Urumqi, China; Central Laboratory, Xinjiang Medical University, Urumqi, China

**Keywords:** phytoestrogens, cold, testicular proteomics

## Abstract

Phytoestrogens and cold negatively influence male fertility. However, the combined effects of these two factors on male reproduction remain unknown. Herein, we studied the changes of sperm parameters and identify potential biomarkers involved in testis of rats, which were intervened by phytoestrogens combined with cold stress. Male Sprague–Dawley rats were randomly divided into control and model groups. The rats in the model group were fed an estrogen diet and placed in a climate chamber [10 ± 2°C; humidity of 75 ± 5%] for 12 h/daily. When compared with the control group after 24 weeks, the rats in the model group showed increased food intake, urine and stool outputs, and higher estradiol and follicle-stimulating hormone levels. However, lower sperm concentration, motility, and viability, and reduced testosterone levels were detected. The epithelial cells of the seminiferous tubules and epididymal ducts presented morphological abnormalities. Proteomic analysis showed that 24 testicular proteins were upregulated and 15 were downregulated. The identified proteins were involved in reticulophagy and stress response. Our findings suggest that the phytoestrogens combined with cold stress had negative effects on the reproductive function of male rats and provide the basis for the establishment of “course simulation” type of oligospermia animal model.

## 1 Introduction

The World Health Organization estimated an approximately 9% of couples suffer from infertility, with the male factor contributing to 50% [1]. Spermatogenesis, which occurs in the seminiferous epithelium in the testis, consists of the differentiation process of the spermatogonia cells to mature spermatozoa. During spermatogenesis and spermatozoon maturation until their combination with the ova, sperm undergoes a series of alterations in morphology and function, each of which is affected by several elements, including genetic, immune, environmental, and neuroendocrine factors. These factors can cause spermatogenic disorders and abnormal sperm motility. Specific proteins in the testis play important physiological roles in sperm generation, maturation, and acquisition. Different pathogenic and environmental factors can cause significant dysregulation of these proteins, thus affecting the spermatogenetic process.

Exposure to environmental estrogen pollution can significantly affect the male reproductive system. Phytoestrogens are natural heterocyclic phenol compounds, which possess structural similarity with estrogen steroids and present estrogenic activity. The classical phytoestrogens, so far known, constitute a group of plant-derived compounds which include mainly isoflavones, lignans, coumestanes, stilbenes, and the flavonoids quercetin and kaempherol. Aliments, such as spinach, bean sprouts, soybeans, cabbage, and hops, contain high levels of phytoestrogens. Several preclinical experiments have demonstrated that the ingestion of high levels of phytoestrogens can negatively affect male reproduction, leading to decreased semen quality and an increase in congenital malformations, such as hypospadias and cryptorchidism. Moreover, phytoestrogens can adversely affect brain differentiation and reproductive development in rodents during embryogenesis [2] and can also increase the incidence of testicular cancer in humans [3].

Cold is one of the strongest physical and psychological environmental stressors, leading to a significant defense response in the body, known as the cold shock response. The body responds to cold shock with long-term adaptive changes in metabolic, physiological, biochemical, and hormonal parameters [4,5,6]. The cold shock causes sympathetic activation and constriction of peripheral blood vessels [7]. Because of their large surface-to-mass ratios, small mammals experience a huge thermoregulatory burden at low temperatures, which in turn induces reproductive inhibition to a certain extent [8,9]. This inhibitory effect of cold on various reproductive parameters has been repeatedly demonstrated in rodents [10].

Xinjiang is located at the following geographical coordinates: 73°40′ E∼96°23′ E, 34°22′ N∼49°10′ N. In this location, the winter season is cold and long. Additionally, environmental pollution has become increasingly serious and estrogen pollutants (both synthetic and naturally derived) are part of the environmental contaminants.

In this study, phytoestrogens combined with cold stress intervention were experimentally reproduced, and changes in sperm parameters in rats were evaluated. Alterations of the testis proteins were detected using label-free proteomic technology. This study aims to set the basis for establishing a “course simulation” type of oligospermia animal model and preliminarily identify its molecular mechanism.

## 2 Methods

### 2.1 Animal models

The experimental animals were bred and provided by the Animal Experiment Center of Xinjiang Medical University. In a 24-week phytoestrogen and cold stress intervention study, 8-week-old Sprague–Dawley rats (180–220 g) were randomly assigned to two groups of 12 males each. Group N served as the control group. Rats in group M were fed phytoestrogens – diet (70% conventional rat diet and 30% spinach seed diet) – and placed, every day for 24 weeks, in a climate chamber [temperature of 10 ± 2°C; humidity at 75 ± 5%] from 9 in the morning to 21 o ‘clock in the evening (total 12 h) [11,12]. Body weight, daily food and water intake, and daily weight of urine and stool were measured before sampling.


**Ethical approval:** The research related to animal use has been complied with all the relevant national regulations and institutional policies for the care and use of animals and in accordance with the Animal Care and Use Committee of Xinjiang Medical University (Res: IACUC-20210302-46).

### 2.2 Sperm analysis

At the end of the experiment, after fasting for 12 h, the rats were anesthetized by intraperitoneal injection of 1% pentobarbital sodium (50 mg/kg). The left epididymis cauda was removed, and sperm suspension was prepared by mincing the tissue with scissors in 4 mL of M199 medium (containing 2% BSA). After incubation at 37°C for 15 min, the tissue fragments were removed from the suspension. Semen analysis was performed according to World Health Organization guidelines (WHO, 2010). The sperm concentration was determined using a Neubauer counting chamber. An aliquot of the sperm suspension was charged into the counting chamber, and spermatozoa were counted. The sperm count was expressed in millions per mL. Sperm motility was determined by placing a drop of 10 µL of the sperm suspension on a 37°C prewarmed slide and covered with a cover slip. At least 200 sperm were assessed for each semen sample using a bright-field microscope, and the percentage of motile sperm was calculated. Sperm viability was assessed using the eosin–nigrosin staining technique, where the dead cells were stained. A sample of 10 µL of sperm suspension was mixed with 0.5 μL of eosin for 30 s, then added 3 μL nigrosin staining solution, and a smear was made on a prewarmed slide. The slides were examined under a microscope with oil immersion (×1,000 magnification) to determine the percentage of viable sperms.

### 2.3 Serum sex hormone level measurement

Blood was collected from the abdominal aorta, and samples were centrifuged at 3,000 × *g* for 15 min at 4°C. The collected serum samples were stored at −80°C. Serum sex hormone levels of testosterone (T), estradiol (E_2_), luteinizing hormone (LH), follicle-stimulating hormone (FSH), and prolactin (PRL) were measured by using radioimmunoassay kits (Beijing North Institute of Biology Technology, Beijing, China) according to the manufacturer’s instructions.

### 2.4 Morphological observation of testis and epididymis

The right testis and epididymis were collected from both experimental groups. The tissue was fixed with 4% paraformaldehyde, embedded in paraffin, sectioned, and evaluated by Periodic Acid-Schiff-hematoxylin (PAS-H) staining and immunofluorescence staining. The left testis was cryopreserved at −80°C for proteomic studies, Western blotting, and quantitative real-time polymerase chain reaction (qRT**-**PCR) analysis.

### 2.5 Proteomic detection

Testicular tissues were lysed in the presence of protease inhibitors, and total protein was extracted. Protein concentration was determined using a bicinchoninic acid (BCA) kit (Solarbio, PC0020, Beijing, China), and protein quality was detected by sodium dodecyl sulfate-polyacrylamide gel electrophoresis (SDS–PAGE). The proteins were hydrolyzed via enzymatic digestion using trypsin. After trypsin digestion, the peptides were resuspended in 25 μL solvent A (A: ACN with 0.1% formic acid), separated by nanoLC, and analyzed by online electrospray tandem mass spectrometry (MS/MS). The experiments were performed on a NanoAquity UPLC system (Waters Corporation, Milford, MA, USA) connected to a Q Exactive hybrid quadrupole Orbitrap-mass spectrometer (Thermo Fisher Scientific, San Jose, CA, USA) equipped with an online nano-electrospray ion source. An aliquot of the peptide sample (8 μL) was loaded onto a Thermo Scientific Ac-claim PepMap C18 column (100 μm x 2 cm, 3 μm particle size), with a flow rate of 10 μL/min for 3 min, and subsequently separated on an analytical column (Acclaim PepMap C18, 75 μm × 15 cm) with a linear gradient from 5% B to 45% B in 75 min. The column was re-equilibrated under initial conditions for 15 min. The column flow rate was maintained at 300 nL/min, and the column temperature was maintained at 40°C. An electrospray voltage of 1.9 kV versus the inlet of the mass spectrometer was used. The Q Exactive mass spectrometer was operated in the data-dependent mode to automatically switch between MS and MS/MS acquisition. Survey full-scan MS spectra (*m*/*z* 300–1,200) were acquired at a mass resolution of 70 K, followed by 15 sequential high-energy collisional dissociation (HCD) MS/MS scans at a resolution of 17.5 K. In all cases, one microscope was recorded using a dynamic exclusion of 30 s. For MS/MS, precursor ions were activated using 27% normalized collision energy.

Comparisons between the two groups were conducted using two-sided unpaired *t*-tests. The selection criteria for differentially expressed testicular proteins were a fold change ≥1.2 or ≤0.8 and a *P*-value < 0.05. All results are expressed as mean ± standard deviation.

### 2.6 Bioinformatics analysis

For functional annotation, the biological process, cellular component, and molecular function of the differentially expressed proteins were analyzed using the Gene Ontology (GO) database. The Kyoto Encyclopedia of Genes and Genomes (KEGG) database was used to identify the pathways that involved target proteins.

### 2.7 Verification

#### 2.7.1 Western blot analysis

Testicular tissues from each rat were separately homogenized and lysed in ice-cold radio-immunoprecipitation assay lysis buffer (Solarbio, R0010, Beijing, China). After centrifugation at 12,000 × *g* for 10 min at 4°C, the supernatants were collected. The protein concentration was determined using the standard BCA kit (Solarbio, PC0020, Beijing, China). Then, 40 µg of each protein sample was loaded and separated by 10% sodium dodecyl SDS–PAGE and transferred to polyvinylidene fluoride (PVDF) membranes (Millipore, Billerica, MA, USA). After blocking in 5% nonfat milk for 2 h at 37°C, the membrane was incubated with the primary antibody overnight at 4°C: integrin alpha 6 (ITGA6) (1:1,000; Boster, BA3356, Wuhan, China), alpha 1 chains of type VI collagen (Col6α1) (1:1,000; Boster, BM4748, Wuhan, China), alpha 2 chains of type VI collagen (Col6α2) (1:500; Affinity, DF3552, Zhenjiang, China), then with a Peroxidase-conjugated AffiniPure Goat Anti-Rabbit IgG(H + L) secondary antibody (1:5,000; ZSGB-BIO, ZB2301, Beijing, China) for 2 h at 37°C. Finally, the membrane was washed with tris base solution-tweet (TBST) and visualized using enhanced chemiluminescence detection (ProteinSimple Flour Chem. E). Afterward, the protein bands were quantified and analyzed using ImageJ software.

#### 2.7.2 Immunofluorescence staining

Paraffin-embedded testicular Section (4 µm thick) were dewaxed with dimethylbenzene and gradient ethanol and then washed with phosphate-buffered saline (PBS). After incubation with 3% hydrogen peroxide, antigen retrieval was performed using 0.01 mol/L sodium citrate buffer (pH 7.0). The sections were then washed thrice with TBST and blocked with goat serum. Primary antibody was incubated overnight at 4°C: ITGA6 (1:200; Boster, BA3356, Wuhan, China), Col6α1 (1:100; Boster, BM4748, Wuhan, China), and Col6α2 (1:100; Affinity, DF3552, Zhenjiang, China). After washing three times, the samples were incubated with a secondary antibody to fluorescein isothiocyanate (FITC)-conjugated anti-rabbit IgG for 30 min at 37°C (1:1,000; abcam, ab150077, Cambridge, UK) and washed three times with TBST; 4′,6′-diamidino-2-phenylindole (DAPI) was used to stain the nuclei of cells in the testis, and the sections were observed for epifluorescence using a Leica Microsystems CMS GmbH microscope.

#### 2.7.3 RNA extraction and qRT-PCR

Testicular tissues from each group (*n* = 10) were randomly selected, and total RNA was isolated using an RNA Easy Fast Animal Tissue Total RNA Extraction Kit (Tiangen, DP431, Beijing, China). Total RNA concentration was measured using a Nanodrop 2,000 spectrophotometer. RNA (1 µg) was then converted to cDNA using a FastKing RT Kit (with gDNase) (Tiangen, KR116-02, Beijing, China) in a 20 μL reaction mixture. Aliquots of 1 μL cDNA samples were subjected to quantitative PCR (qPCR) in a total volume of 10 μL using SuperReal PreMix Plus (SYBR Green) (Tiangen, FP205, Beijing, China) in a QuantStudio 6 Flex. The primers used were GAPDH F-TGAATACGGCTACAGCAACAGG, R-GTGAGGGAGATGCTCAGTGTTG, ITGA6 F-CGCCGCCGCTCAGAATATCAAG, and R-CAAGCACAGCCAGGAGGATGATC, Col6α1 F-CCTCCTGGTCCCTCCCTCTTTG, R-TTGTCTCTGCTTGCTGCCGTTAC, Col6α2 F-TCCTCCTCCTCTTCCTCCTCCTC, R-AGCAAGTTCCAGAACAGTCAAGGC. GAPDH mRNA was used as an internal control.

### 2.8 Statistical analyses

Comparison between experimental groups was performed by the independent two-tailed *t*-test using SPSS version 26.0 software (IBM), and *P <* 0.05 was considered significant.

## 3 Results

### 3.1 Estrogen-enriched diet and cold shock reduced spermatogenesis

To determine the effect of estrogen pollution combined with cold shock, 12 rats were exposed to a diet rich in estrogen and placed for 12 h daily at a lower temperature (10 ± 2°C) for 24 weeks (group M) and were compared with a control group (N, including 12 rats). Body weight, daily food and water intake, and urine and stool weights were measured. Compared with the control group, no apparent changes in the final body weight were observed. However, significant increase in daily food intake, weight of urine output, and stool output as well as reduction in water intake were detected (*P* < 0.05, Table 1).

**Table 1:** Body weight, testis and epididymis weight, daily food and water intake, and daily weight of urine and stool

Group Parameters	Group N	Group M
Body weight (g)	625.25 ± 47.48	619.00 ± 69.43
Food intake (g)	37.00 ± 11.47	47.91 ± 5.39**
Water intake (mL)	50.83 ± 12.99	40.00 ± 4.82*
Weight of urine (g)	13.08 ± 3.92	27.75 ± 3.51***
Testis (g)	1.76 ± 0.21	1.39 ± 0.49*
Epididymis (g)	0.84 ± 0.10	0.62 ± 0.16**
Testis index	0.002822 ± 0.000357	0.002206 ± 0.000649**
Epididymis index	0.001347 ± 0.000213	0.000998 ± 0.000259**

Values are expressed as mean ± SD. Statistical significance is indicated as * P < 0.05, ** P < 0.01, and *** P < 0.001 in comparison with control group.

Note: Testis index = Testis Weight/Body Weight. Epididymis index = Epididymis Weight/Body Weight.

**Figure 1:**
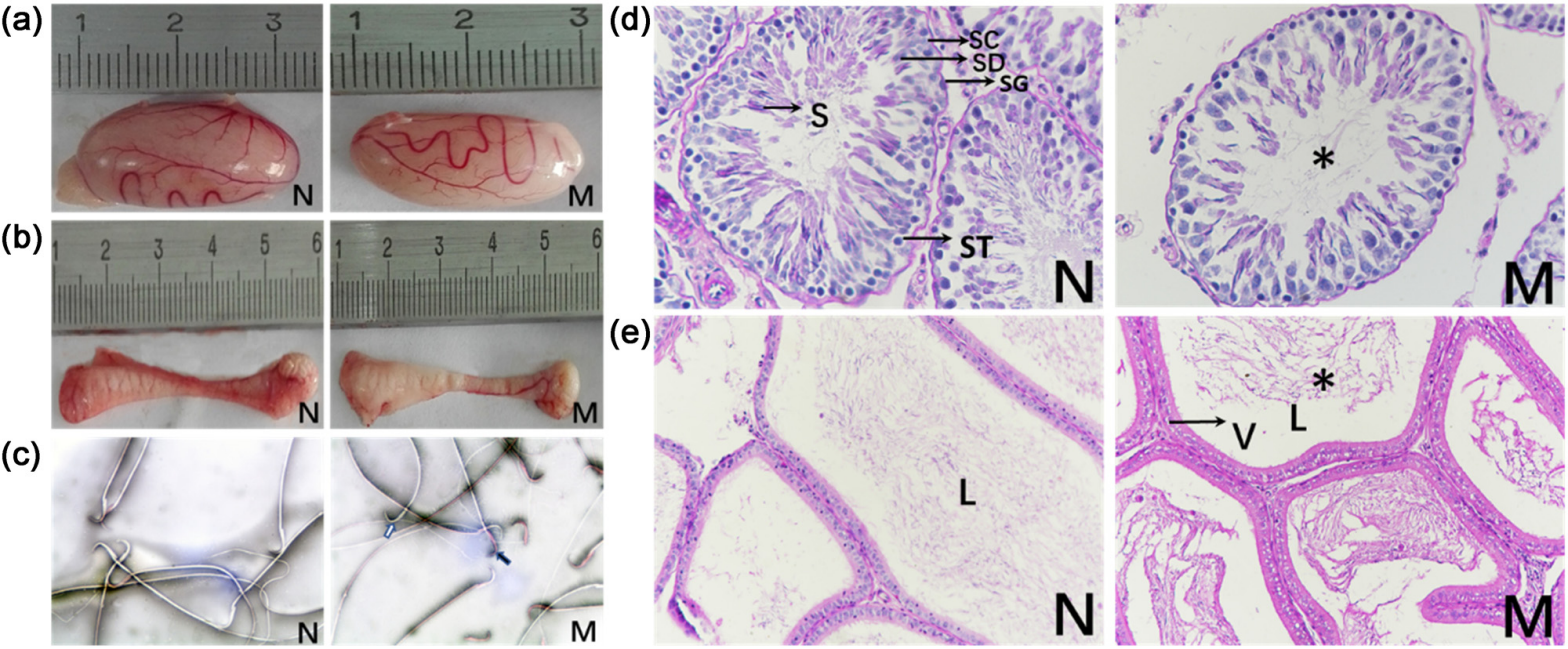
Effects of phytoestrogens combined with cold stress on the sperm viability and morphology of testis and epididymis in rats. At the end of the 24-week treatment, right testes, epididymis, and sperm were collected from 12 rats exposed to estrogen-enriched diet and cold (group M), and 12 rats control (group N). (a) Size of testis. (b) Size of epididymis. (c) Eosin/nigrosin staining of sperms of rats in each group (×1000). The staining was used to define sperm viability. Stained sperms were dead (black arrows), unstained were alive (white arrows). (d) Histological evaluation of testis by Periodic Acid–Schiff–hematoxylin (PAS-H) staining (×400). Spermatogonia (SG), primary spermatocyte (ST); spermatid (SD); early-stage sperm (S); reduced sperm density (*). (e) Histological evaluation of epididymis by Periodic Acid–Schiff–hematoxylin (PAS-H) staining (×200). Group M showed increased vacuoles in the cytoplasm (V) and reduced sperm density (*) in the lumen (L).

Moreover, tissue analysis revealed that the weight of the testis and epididymis decreased dramatically compared with the control group (*P* < 0.05, Table 1). The size of the testis and epididymis is shown in Figure 1a and b. Furthermore, evaluation of the sperm quality parameters showed that compared with control group (N), sperm concentration (*P* < 0.01), motility (*P* < 0.001), and viability (*P* < 0.05) in the model group (M) were significantly lower (Table 2). The eosin/nigrosin staining is shown in Figure 1c.

**Table 2:** Sperm quality parameters measured in group N and M

Sperm quality parameters	Group N	Group M
Sperm concentration (10^6^/mL)	62.53 ± 14.09	45.19 ± 11.94**
Motility (%)	62.42 ± 4.33	39.94 ± 11.10***
Viable sperm (%)	80.12 ± 8.23	68.64 ± 16.08*

Values are expressed as mean ± SD. Statistical significance is indicated as * *P <* 0.05, ** P < 0.01, and *** P < 0.001 in comparison with the control group.

### 3.2 Changes in serum sex hormone levels

To define the effect of exposure to phytoestrogens and cold on physiological reproductive parameters of the rats, serum sex hormone levels of testosterone (T), estradiol (E_2_), LH, FSH, and PRL were measured. Compared with group N, T levels in group M were significantly decreased, whereas E_2_ and FSH levels were increased (*P <* 0.05). However, no significant change in LH and PRL levels was observed (Table 3).

**Table 3:** Serum sex hormone levels

	Group N	Group M
T (ng/mL)	1.67 ± 0.79	0.47 ± 0.30***
E_2_ (µIU/mL)	37.97 ± 6.15	49.68 ± 16.72*
LH (mIU/mL)	4.57 ± 1.60	5.64 ± 2.29
FSH (mIU/mL)	5.10 ± 2.82	11.05 ± 6.46**
PRL (pg/mL)	84.49 ± 41.82	102.54 ± 37.83

Values are expressed as mean ± SD. Statistical significance is indicated as * P < 0.05, ** P < 0.01, and *** P < 0.001 in comparison with the control group.

### 3.3 Morphological variations of testis and epididymis

Histological analysis of the testes revealed that the spermatogenic tubules in group N were abundant, various levels of spermatogenic cells were neatly arranged into 5–8 layers, and Sertoli cells were visible. However, the number of spermatogenic cells in group M was significantly lower than that in group N (Figure 1d). Moreover, the epididymis duct showed vacuolar denaturalization of the cytoplasm in the epididymal epithelium. The lumen contained fewer sperm in group M (Figure 1c).

### 3.4 Differentially expressed testicular proteins

**Figure 2:**
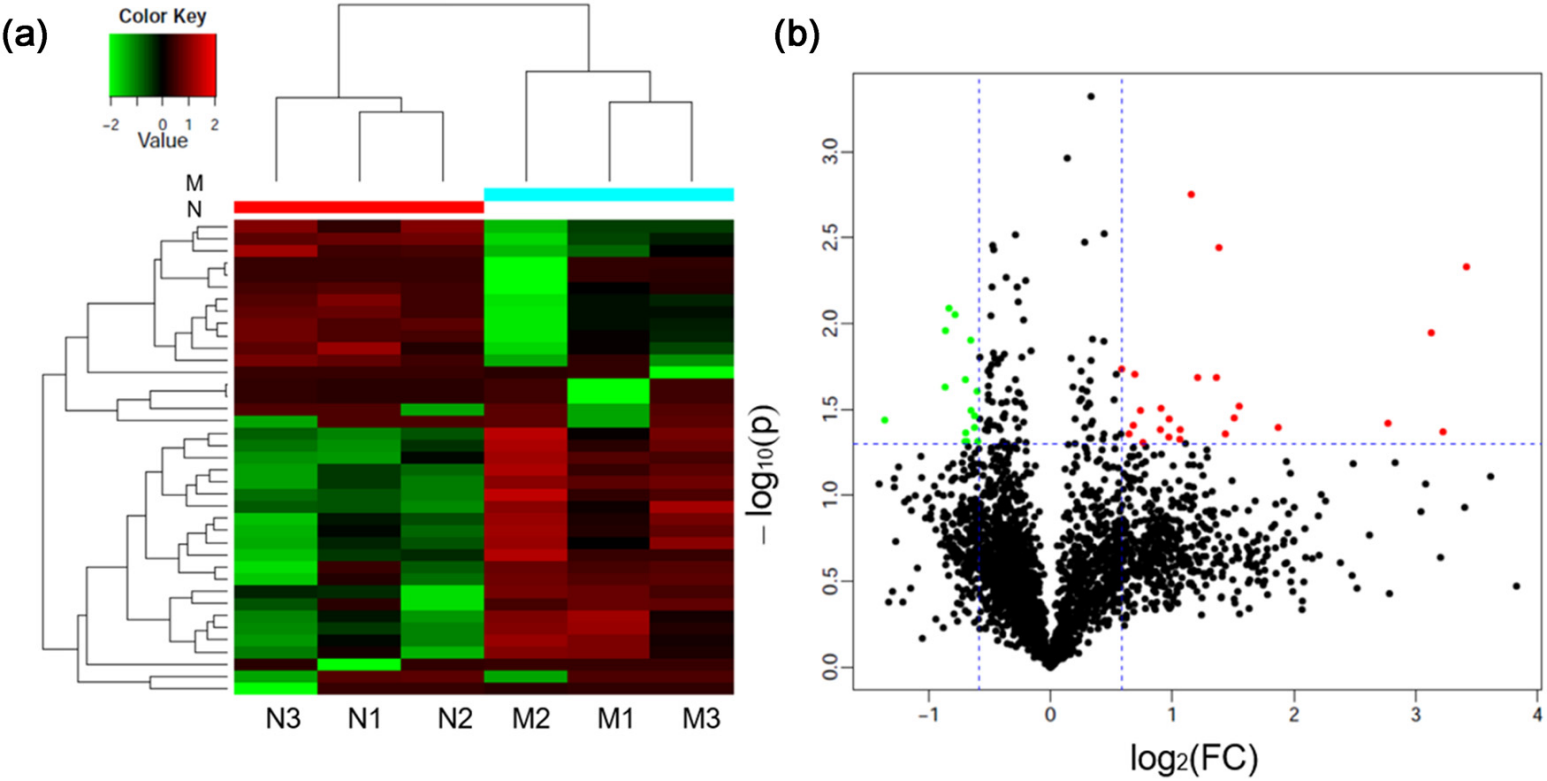
Altered expression of proteins in testis exposed to estrogen-enriched diet and cold for 24 weeks. (a) Volcano plot showing proteins differentially expressed between control group (Group N) and model group (Group M). In the volcano plot, the *X*-axis represents the log2 (fold-change (FC)) and the *Y*-axis is the *P* value. Data points in the upper right (FC > 2.0) and upper left (FC < 0.5) with *P* < 0.05 represent proteins that significantly altered. (b) Heatmap of proteins with altered abundance in testis. Rows represent the individual samples. Red color represents upregulated proteins and the green color represents downregulated proteins. Black indicates no difference in protein quantity.

**Figure 3:**
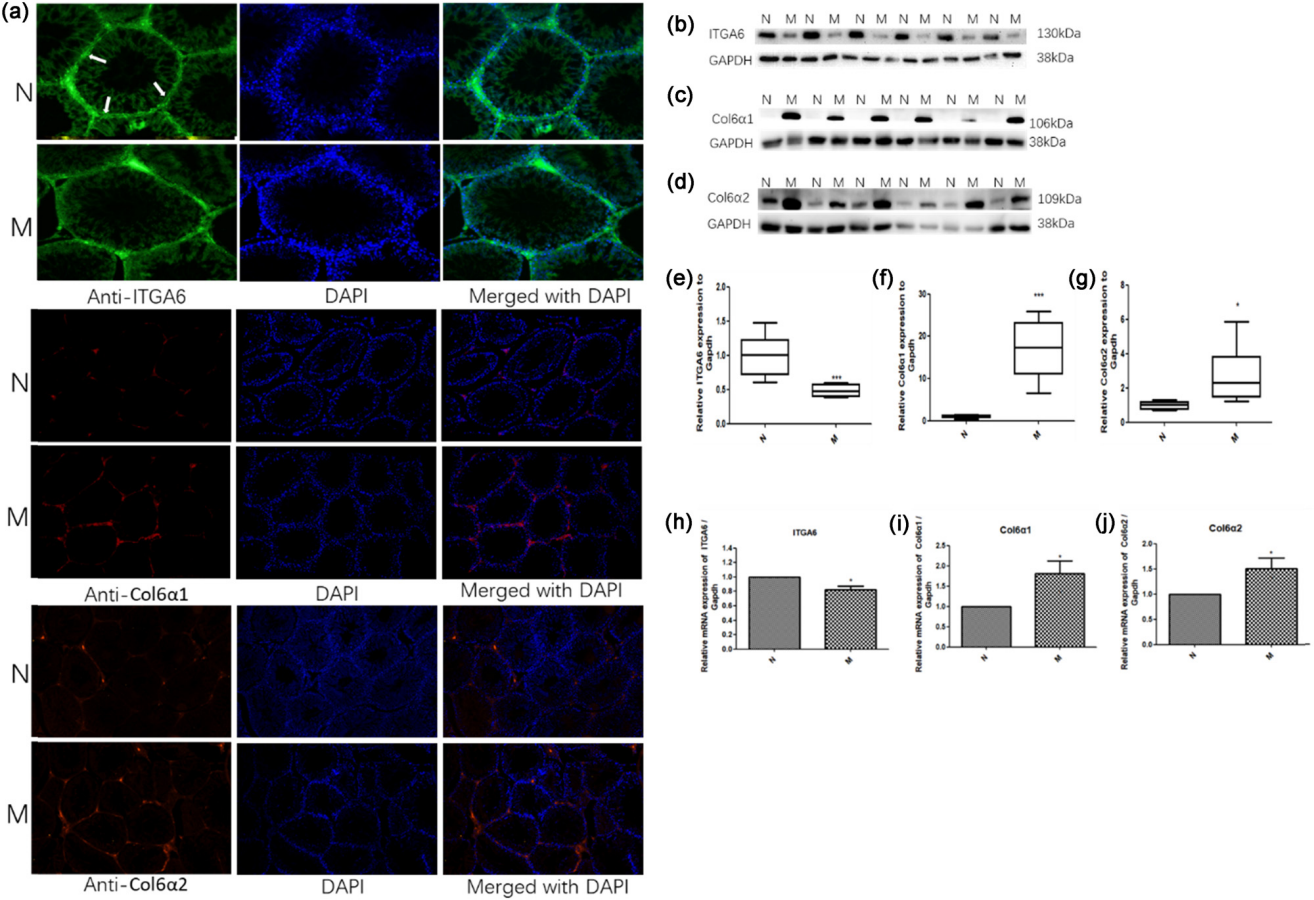
ITGA6 was downregulated, Col6α1 and Col6α2 were upregulated in the testis of the model group exposed to an estrogen-enriched diet and cold for 24 weeks. (a) Immunofluorescence staining for ITGA6 (red, 200×), Col6α1 (green, 100×), Col6α2 (red, 100×); nuclei were stained with DAPI (blue). (b, c, and d) Representative western blot results for ITGA6, Col6α1, Col6α2. (e, f, and g) Relative expression of ITGA6, Col6α1, Col6α2 compared with GAPDH. Protein lysates were obtained from the testis of either model (M) or control rat group (N). Protein expression of ITGA6, Col6α1, and Col6α2 was evaluated by western blotting using specific antibodies for the target proteins, and the signal was then quantified using Image J. (h, i, and j) qRT-PCR of ITGA6, Col6α1, and Col6α2 in the testis of rats in the two groups. Values are expressed as the mean ± SD. Statistical significance is indicated as * *P* < 0.05, ** *P* < 0.01, and *** *P* < 0.001 compared to the control group.

Proteomic analysis of testis tissues from N and M groups was performed, and 4,750 proteins were extracted from 32,479 unique peptides. A total of 3,455 proteins were common between the two groups. Moreover, 24 proteins were upregulated and 15 downregulated in group M. The top ten upregulated proteins, based on the highest fold change (FC), included alpha 5 chains of type VI collagen (Col6α5, FC = 10.7, *P* = 0.005), H1.2 linker histone (FC = 9.3, *P* = 0.04), alpha 1 chain of type VI collagen (Col6α1, FC = 8.7, *P* = 0.01), alpha 2 chain of type VI collagen (Col6α2, FC = 6.8, *P* = 0.04), 40 S ribosomal protein S6-like (FC = 3.6, *P* = 0.04), immunoglobulin heavy constant mu (FC = 2.9, *P* = 0.03), macroH2A.1 histone (FC = 2.8, *P* = 0.03), sorbin and SH3 domain-containing 2 (FC = 2.4, *P* = 0.04), B-cell receptor-associated protein 31 (FC = 2.6, *P* = 0.004), and transgelin (FC = 2.6, *P* = 0.02). The top ten downregulated proteins included the hypothetical LOC287798 (FC = 0.4, *P* = 0.04), translocase of the outer mitochondrial membrane 70,3′(2′) (FC = 0.5, *P* = 0.02), 5′-bisphosphate nucleotidase 1 (FC = 0.5, *P* = 0.01), structure-specific recognition protein 1 (FC = 0.6, *P* = 0.008), dynein light chain LC8-type 1 (FC = 0.6, *P* = 0.009), coiled-coil domain containing 58 (FC = 0.6, *P* = 0.04), doublecortin domain containing 2 C (FC = 0.6, *P* = 0.02), similar to 60 S ribosomal protein L38 (FC = 0.6, *P* = 0.04), TOR signaling pathway regulator, and metallothionein 3 (FC = 0.6, *P* = 0.04, Figure 2). The upregulated proteins Col6α1, Col6α2, and downregulated proteins integrin alpha 6 (ITGA6) were validated by Western blotting, immunofluorescence, and real-time quantitative reverse transcription (qRT-PCR; Figure 3).

### 3.5 GO enrichment and pathway analyses

**Figure 4:**
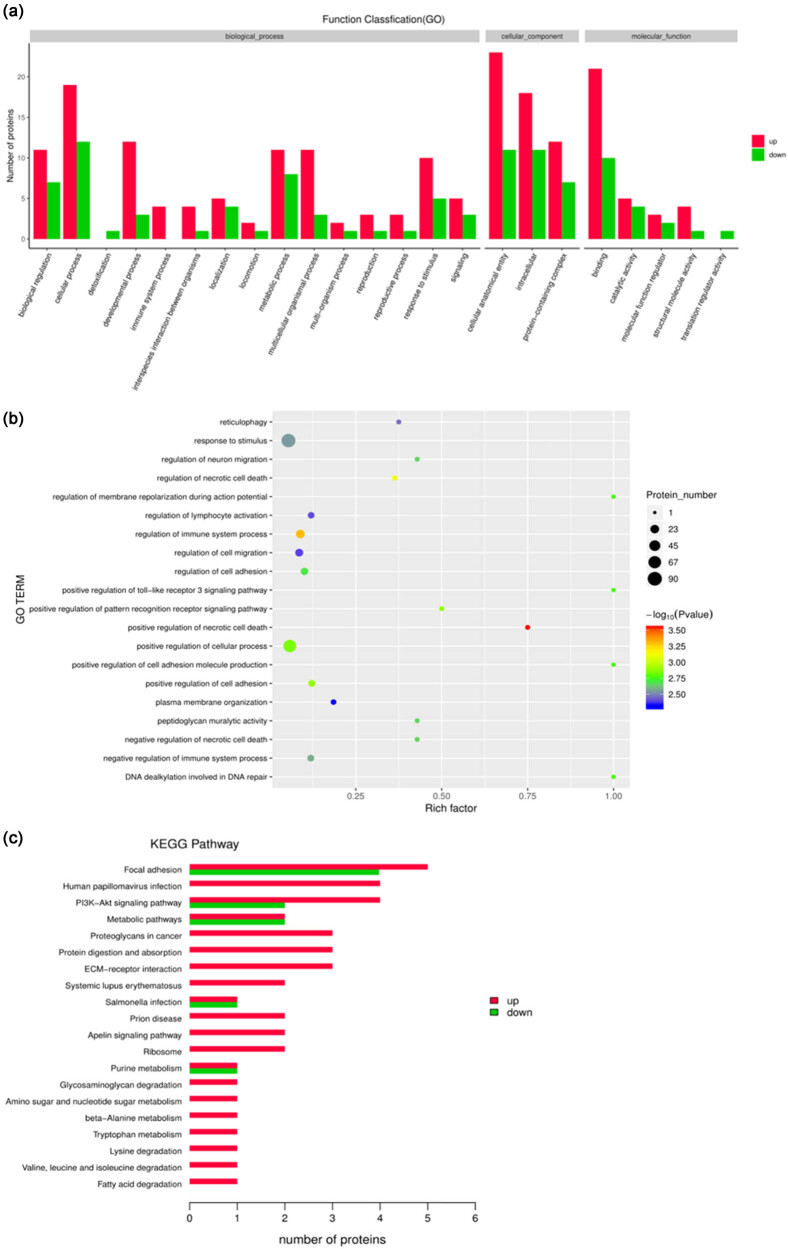
Effects of phytoestrogens combined with cold stress on testicular proteomics in rats. (a) All enriched gene ontology (GO) terms of the proteins between the model group and the control. The enriched terms of molecular function, cellular component, and biological process categories were identified. (b) GO functional significance enrichment analysis. (c) The enriched Kyoto encyclopedia of genes and genomes (KEGG) pathways of proteins between the model group and the control.

Gene Ontology (GO) enrichment analysis was performed to classify the biological processes, cellular components, and molecular functions of these proteins (Figure 4a). The differentially expressed proteins were mainly involved in reticulophagy, response to stimulus, regulation of neuron migration, regulation of necrotic cell death, regulation of membrane repolarization during action potential, regulation of lymphocyte activation, regulation of immune system processes, regulation of cell adhesion, cell migration, and positive regulation of the toll-like receptor 3 signaling pathway (Figure 4b).

Pathway analysis of the differentially expressed proteins was performed using the KEGG. The top five pathways containing the largest number of proteins were focal adhesion (FAs), human papillomavirus infection, PI3K-Akt signaling pathway, metabolic pathway, and cancer proteoglycan (Figure 4c). Specifically, three upregulated proteins (Col6α5, Col6α1, and Col6α2) and two downregulated proteins (integrin subunit ITGA6 and receptor tyrosine kinase (RTK)) were involved in the PI3K-Akt signaling pathway. Moreover, four upregulated proteins (Col6α1, Col6α2, caveolin, and filamin) and three downregulated proteins (ITGA6, Rho GTPase activation protein (RhoGAP)), and Parvin were involved in the FA signaling pathway included.

## 4 Discussion

Spermatogenesis in mammals is a cyclic process of spermatogenic cell development occurring in the seminiferous tubules. The formation, maturation, and capacitation of spermatogenic cells are susceptible to dietary changes and environmental factors. Temporary sterility syndrome in livestock (ewes) is associated with high phytoestrogen intake during grazing [13]. Humans and rodents consume high doses of phytoestrogens, which can cause decreased testicular weight, sperm count, and motility [14]. In this study, the rats in the model group showed indeed these listed phenotypes. Androgen plays an important role in testicular development and sperm parameters, thus decreased testicular weight and sperm parameters might be linked to the reduction in serum testosterone caused by phytoestrogens [15].

Temperature is an important environmental factor affecting the metabolism and reproduction of small mammals [16]. The inhibition of reproductive function in winter occurs commonly among small mammals living in temperate zones [17]. In some rodents, it has been shown that cold exposure not only could suppress growth but also delay the onset of breeding [18]. In this study, the model rats that were placed in a cold environment showed that food intake, weight of urine output, and stool output significantly increased, whereas body weight continued to slowly grow. It has been reported that animals increase energy intake and thermogenesis to maintain energy balance in order to respond to cold conditions. They might allocate less energy to the development of reproductive organs [19].

Morphological study of the testis and epididymis showed that the epithelial cells of seminiferous tubules and ductus epididymis were obviously different between the control and model groups. Compared with group N, the number of sperm in the lumen of the epididymis decreased dramatically. The changes in hormone levels were responsible for abnormal morphology. Serum sex hormone results showed that the testosterone level in the model group significantly decreased, whereas the E_2_ and FSH levels increased after treatment with phytoestrogens combined with cold stress. This is related to the competitive binding of phytoestrogens to the estrogen receptor, which inhibited its negative feedback regulation, promoted the positive feedback regulation of LH secretion, and stimulated the secretion of E_2_. FSH is essential for the initiation of spermatogenesis, but the abnormal increase of serum FSH level often means that the seminiferous epithelium has been damaged. At the same time, when testicular cells were exposed to a high-estrogen environment, the expression of steroid synthase decreased, which could also lead to a further decrease in T level [20]. In addition, long-term cold stimulation can cause a chronic stress response that disturbs the balance of hormones and eventually results in reproductive damage.

Further proteomic studies identified 39 testicular proteins differentially expressed between the two experimental groups. Among these proteins, integrin α6 (ITGA6) and collagen6 (chain α1 (Col6α1) and chain α2 (Col6α2)) were detected with a high fold-change. Integrins play a key role in cell adhesion to extracellular matrix (ECM) ligands and adjacent cells and serve as links between extracellular contacts and the intracellular cytoskeleton. In addition, integrins work together with receptor tyrosine kinases to communicate bidirectional signals between cells and ECM [21]. They are important for the regulation of cell proliferation, differentiation, survival, and migration of cells [22]. ITGA6 is a spermatogonia-specific marker and receptor subunit of laminin that participates in the formation of the seminiferous tubule basement membrane and spermatogonial stem cells (SSCs). Spermatogenesis was reconstituted in infertile male mice after colonization assays in recipient testes using testis cells expressing ITGA6 [23]. Additionally, ITGA6 plays an essential role in the PI3K/AKT pathway, which is closely related to spermatogenic function. So, the low expression of ITGA6 is responsible for the decreased number of germ cells in the testes of group M. Col6 is a major ECM protein that forms a microfibrillar network in many tissues, including skeletal muscle, cartilage, tendon, lung, nervous system, adipose tissue, skin, eye, heart, and vasculature [24]. This protein is composed of three major chains: α1(VI), α2(VI), and α3(VI). Biosynthesis of type VI collagen requires these three chains to be assembled intracellularly into monomers, dimers, and tetramers, which are then secreted and beaded into extracellular microfilaments and then deposited in the ECM [25,26]. Recently, three new chains similar to α3(VI) were discovered: α4(VI), α5(VI), and α6(VI) [27]. Their distribution is limited to specific tissues, such as skeletal muscles, cardiac muscles, and reproductive organs. Testicular collagen consists of several types of collagen and is thought to play a significant role in the structural organization of seminiferous tubules and interstitial tissues. Sertoli cells and SSCs attach to the basement membrane in the seminiferous epithelium. Moreover, pathological studies have shown that the basement membrane of the seminiferous tubule is thickened and the interstitial tissue is increased in male infertility patients, children with cryptorchidism, and prostate cancer patients [28,29,30]. It has been indicated that the collagen content was increased in the testes of such patients [31]. Although its specific function in the testes has not yet been reported, in this study, the expression of Col6α1 and Col6α2 were all increased in the basement membrane of the seminiferous tubule in group M, and the upregulated Col6 would negatively affect the attachment of spermatogenic cells and sertoli cells to the basement membrane, then affecting the sperm spermatogenesis and sperm capacitation. Further research is needed to reveal this mechanism.

GO enrichment analysis showed that the differentially expressed proteins were mainly involved in reticulophagy and response to stimulus. The endoplasmic reticulum (ER) is a biosynthetic organelle in eukaryotic cells. Its capacity to produce proteins, lipids, and oligosaccharides responds to physiological and pathologic demands [32]. Reticulophagy refers to the increase in unfolded or misfolded proteins in the ER when the internal and external environments of cells change, leading to an ER stress response. Changes in ER function disrupt cellular homeostasis and then activate selective autophagy to remove damaged endoplasmic reticulum or segments in cells. Overactivated reticulophagy in testicular tissue can also remove normal endoplasmic reticulum structures and luminal fragments from spermatogenic cells, resulting in spermatogenesis disorders.

Pathway analysis showed that the differentially expressed proteins were involved in 51 signaling pathways, among which the PI3K-Akt signaling pathway, FA signaling pathway, metabolic pathway, protein digestion, and absorption pathways involved more proteins. Several studies have shown that the PI3K/Akt pathway plays an essential role in the self-renewal of SSCs and the differentiation of spermatogonia [33]. In Akt−/− mice, apoptotic sperms increased [34]. In addition, the PI3K/Akt signaling pathway has protective effects and contributes to the survival of germ cells in mice after toxic testicular injury. In asthenospermia, changes in the expression of PI3K/AKT and cAMP-mediated PKA signaling pathways lead to flagellin phosphorylation and abnormal sperm motility [35,36,37]. Moreover, the PI3K-Akt signaling pathway is related to FSH regulation of the proliferation process in testicular cells [38]. In this study, the abnormal FSH levels, the decrease in spermatogenic cells, and poor sperm motility might be due to deregulated PI3K/Akt pathway. Integrins are the major transmembrane receptors at sites of cell adhesion to the ECM. The ECM, in turn, is linked to the actin cytoskeleton via peripheral proteins. These specialized attachments and signaling complexes are known as FAs [39]. FA signaling is linked with cell proliferation, differentiation, and cell activity. It regulates gene expression and cell survival and participates in the construction of cell membrane acceptors and the actin cytoskeleton [40]. During sperm capacitation, actin aggregation and cytoskeleton remodeling require focus-binding kinases [41,42,43]. Cell adhesion permits the attachment of developing germ cells to Sertoli cells and the basement membrane in the seminiferous epithelium. All aforementioned findings suggested that the deregulated FA pathways would have led to the decrease of spermatogenic cells and abnormal shedding of germ cells. Proteins involved in both PI3K/AKT and FA signaling pathways included ITGA6, which we found downregulated, and α1 and α2 chains of type VI collagen, upregulated in the model group. These three proteins may, therefore, represent potential biomarkers for oligospermia.

## 5 Conclusion

Rats treated with phytoestrogens combined with cold stress showed higher food intake, increased urine and stool output, and lower sperm concentration, motility, viability, and testosterone level. The epithelial cells of seminiferous tubules and epididymis presented abnormal morphology. Moreover, ITGA6 downregulation and upregulation of alpha 1 and alpha 2 chains of type VI collagen in the testis suggest that alterations of PI3K/AKT and FA pathways might represent the molecular mechanism behind the estrogen/cold-induced infertility. Therefore, this study lays the foundation for the establishment of “course simulation” type of oligospermia animal model.

## Supplementary Material

Supplementary Table
