# Supplementary material for: Effects of phytoestrogens combined with cold stress on sperm parameters and testicular proteomics in rats
Source: Open Life Sci. 2023 Jan 24;18(1):20220531. doi: 10.1515/biol-2022-0531 (PMC9883686; doi:10.1515/biol-2022-0531)
Supplement: Supplementary Table [file biol-2022-0531-sm.pdf]

Supplementary material

Table S1: Differentially expressed proteins in testis

Accession	Description	Score	Sequence coverage [%]	FC	*P*-value
ENSRNOP00000068883.1	collagen type VI alpha 5 chain	323.31	20.3	10.67	0.005
ENSRNOP00000073681.1	H1.2 linker histone	30.254	23.1	9.34	0.043
ENSRNOP00000001679.4	collagen type VI alpha 1 chain	323.31	28.7	8.75	0.011
ENSRNOP00000001695.4	collagen type VI alpha 2 chain	224.56	17.5	6.82	0.038
ENSRNOP00000010383.5	40S ribosomal protein S6-like	25.869	15.8	3.66	0.040
ENSRNOP00000075175.1	immunoglobulin heavy constant mu	8.5875	33.8	2.93	0.030
ENSRNOP00000015632.4	macroH2A.1 histone	37.311	29.6	2.85	0.035
ENSRNOP00000029182.4	sorbin and SH3 domain containing 2	14.084	8.9	2.71	0.044
ENSRNOP00000070305.1	B-cell receptor-associated protein 31	9.3765	18	2.61	0.004
ENSRNOP00000024030.3	transgelin	133.82	49.8	2.57	0.021
ENSRNOP00000054780.4	Ig lambda-2 chain C region	296.88	65.4	2.31	0.021
ENSRNOP00000076288.1	nipsnap homolog 2	5.2638	11.7	2.23	0.002
ENSRNOP00000070076.1	adenylate kinase 3	137.56	40.1	2.09	0.042
ENSRNOP00000073712.1	caveolin 1	4.741	13.5	2.09	0.047
ENSRNOP00000070703.1	filamin A	323.31	41	1.97	0.036
ENSRNOP00000028363.5	double PHD fingers 2	5.1239	6.1	1.96	0.046
ENSRNOP00000073101.1	actin alpha 2, smooth muscle	323.31	72.3	1.88	0.031
ENSRNOP00000061381.3	cytochrome b5 reductase 3	67.838	43.2	1.87	0.041
ENSRNOP00000075883.1	microtubule-associated protein 2	12.527	10.6	1.69	0.049
ENSRNOP00000002410.2	enoyl-CoA hydratase and 3-hydroxyacyl CoA dehydrogenase	5.9219	7.8	1.67	0.032
ENSRNOP00000014860.5	arylsulfatase B	5.362	5.7	1.62	0.020
ENSRNOP00000025534.7	complement C5	15.811	5.1	1.60	0.040
ENSRNOP00000011991.3	cysteine and glycine-rich protein 1	323.31	50.8	1.56	0.044
ENSRNOP00000061250.1	ribosomal protein S11	5.4871	22.8	1.50	0.018
ENSRNOP00000036568.4	RB binding protein 4, chromatin remodeling factor	40.792	27.5	0.66	0.048
ENSRNOP00000002075.3	integrin subunit alpha 6	2.4129	8.4	0.66	0.025
ENSRNOP00000002052.3	RAB4B, member RAS oncogene family	17.901	34.7	0.65	0.041
ENSRNOP00000027986.5	eukaryotic translation initiation factor 3, subunit G	145.16	30.6	0.65	0.034
ENSRNOP00000056268.3	capping protein regulator and myosin 1 linker 1	38.679	12.6	0.64	0.032
ENSRNOP00000025669.3	metallothionein 3	8.1876	47	0.64	0.012
ENSRNOP00000004083.5	TOR signaling pathway regulator	14.021	14	0.62	0.049
ENSRNOP00000050353.2	similar to 60S ribosomal protein L38	27.024	50	0.62	0.043
ENSRNOP00000011048.6	doublecortin domain containing 2C	21.916	19.4	0.62	0.021
ENSRNOP00000047028.3	coiled-coil domain containing 58	41.147	52.8	0.61	0.049

(*Continued*)

Table S1: *Continued*

Accession	Description	Score	Sequence coverage [%]	FC	*P*-value
ENSRNOP00000061342.1	dynein light chain LC8-type 1	51.279	49.4	0.58	0.009
ENSRNOP00000073084.1	structure specific recognition protein 1	49.793	26.4	0.56	0.008
ENSRNOP00000071594.1	3'(2'), 5'-bisphosphate nucleotidase 1	71.414	39.9	0.55	0.011
ENSRNOP00000002238.5	translocase of outer mitochondrial membrane 70	60.078	26.8	0.55	0.023
ENSRNOP00000029802.3	hypothetical LOC287798	99.639	28.2	0.39	0.037
